# Photonic-plasmonic hot-electron-based photodetection with diffracted-order-resolved leaky plasmonic mechanisms

**DOI:** 10.1515/nanoph-2022-0370

**Published:** 2022-08-19

**Authors:** Yin-Jung Chang, Ko-Han Shih, Chun-Yu Hsiao

**Affiliations:** Department of Optics and Photonics, National Central University, Taoyuan City, Taiwan; CREOL, The College of Optics and Photonics, University of Central Florida, Orlando, FL 32816, USA; Department of Optics and Photonics, National Central University, Taoyuan City, Taiwan

**Keywords:** hot electron, leaky wave, metal grating, photodetector, plasmonics

## Abstract

Although hot-carrier-based photodetection using plasmonic effects has been widely investigated, photodetectors of this type with an external quantum efficiency (EQE) >1% and an active area of <1 mm^2^ remain out of reach even in the visible frequencies. In this work, a novel hot-electron-based, non-trench-type photodetector exploiting pure photoexcitation in a thin aluminum (Al) film and leaky plasmonic modes at and between its heterojunctions is proposed, analyzed, and experimentally demonstrated. Combining diffracted-order-resolved analytical analysis and numerical computations unravels the optical absorption mechanism of the innovative design. Leaky surface plasmon resonance (with leakage radiation into the air) produced by a propagating diffracted order and quasibound supermodes (with power leakage via coupled gap plasmon polariton and bound surface plasmon polariton modes) excited by evanescent diffracted orders are shown to significantly contribute to the absorptance in the preferred thin Al film where hot electrons are generated. At 638.9 nm and electric bias −0.9951 V, the measured per-unit-area responsivity, detectivity, and the external quantum efficiency reach 298.1444 μA/mW/mm^2^, 4.3809 × 10^9^ cm Hz^1/2^/W, and 2.6878%, respectively, from an active area of 4.6457 × 10^−2^ mm^2^. The performance is among the best of those previously reported operating at similar wavelengths and biases. The *RC* time constant is estimated to be about 1.673 μs from the current-voltage measurements. The physical insight into the innovative, experimentally demonstrated device could lay the groundwork for the practical use of low-voltage, metal-based photodetection.

## 1 Introduction

Hot-carrier-based photon energy conversion has been the subject of exciting, rather extensive, and sustained research for more than one decade. Fields of research include photovoltaics [1], [2], [3], photocatalysis [4], photoelectrochemical cell [5], and photodetection [6], [7], [8], [9], [10], [11], [12], [13], [14], [15], [16], [17], [18], [19], [20], [21] in both visible and near infrared regimes. Recently, fundamental limits of hot carrier injection from metal has also been discussed [22]. In various attempts to harvest photons, plasmonic nanostructures are essential to all processes ranging from light absorption via localized surface plasmon resonance, giving rise to intense, highly localized electromagnetic fields, to hot electron generations via nonradiative plasmon decay, producing hot electrons via intraband transitions. On the other hand, electrons with energies higher than the Fermi level can be excited optically via interband transitions where electrons from below the Fermi level are excited to higher energy levels in other bands (e.g. from *d* bands to the *sp* conduction band for noble metals), though such transitions also take place in non-radiative decay during plasmon resonance [23, 24].

Among various hot-carrier-based Schottky photodetectors, gold (Au) and silver (Ag) are the two most commonly used plasmonic noble metals, whereas aluminum (Al) remains much less explored at visible and infrared frequencies. While a hot-hole-based plasmonic photodetector (PD) using an ultrathin nanostructured Al film has demonstrated a peak responsivity of ∼4.5 mA/W at a free-space wavelength *λ*_0_ = 1304 nm [20], other attempts to realize plasmonic photodetection with Al at visible frequencies show a responsivity of only a few nA/W [15] to 360 μA/W with Au-plated nanocone array [16]. Because of its relatively high bulk plasma frequency, Al exhibits a broad frequency range from ultraviolet to infrared regimes in which surface plasmon polaritons (SPPs) are sustained at a simple Al/air interface. In the meantime, and perhaps equally important, photoexcited electrons via vertical interband transitions can also occur in Al over a broad frequency range [25] similar to its plasmonic response. Since photon energies of visible light are higher and bulk Al has a larger density of states for energies above the Fermi energy (when compared to that of Au, Ag, and copper), Al-based Schottky photodetection is expected to exhibit a higher per-unit-area responsivity than other plasmonic noble metals in the visible spectrum. Yet such a prediction has not been proved up to the present time.

On the other hand, it is worth mentioning that the larger the active region (where photon energy conversion takes place), the larger the number of states into which hot electrons can be excited, thus increasing the photocurrent. As a result, enlarging the active area of hot-electron-based PDs is a common measure to increase the responsivity, in particular when the conversion efficiency is known to be extremely low. However, a large active area often translates to a larger *RC* time constant and would eventually limit the temporal response of such devices.

In this work, Al-based photon energy conversion with an innovative structure exploiting photonic and leaky plasmonic waves is theoretically investigated and experimentally demonstrated. Diffracted-order-resolved macroscopic physical processes are understood using an exact rigorous electromagnetic formulation in conjunction with numerically-obtained modal fields, unraveling quasi-bound supermodes and leaky surface plasmon resonance that are responsible for absorption enhancement in the preferred planar-Al film where hot electrons are generated. The fabricated Al-based, photonic-plasmonic PD has an active area of approximately 4.6457 × 10^−2^ mm^2^, as opposed to about 1 mm^2^ or above commonly seen in the literature (e.g. 1 mm^2^ in [3], 0.28 cm^2^ in [11], 28 mm^2^ in [18], about 127 mm^2^ in [16], 6.25 cm^2^ in [17], and 9 cm^2^ in [13]), yet the measured per-unit-area responsivity reaches a record high among those operating at similar wavelengths and bias voltages. The detectivity quantified using the measured responsivity and dark current is close to 4.4 × 10^9^ cm Hz^1/2^/W, despite a small active area compared to those previously reported. The *RC* time constant is also estimated based on the measurement results to assess the potential temporal response of the present PD.

## 2 The proposed photonic-plasmonic Schottky photodetector

**Figure 1:**
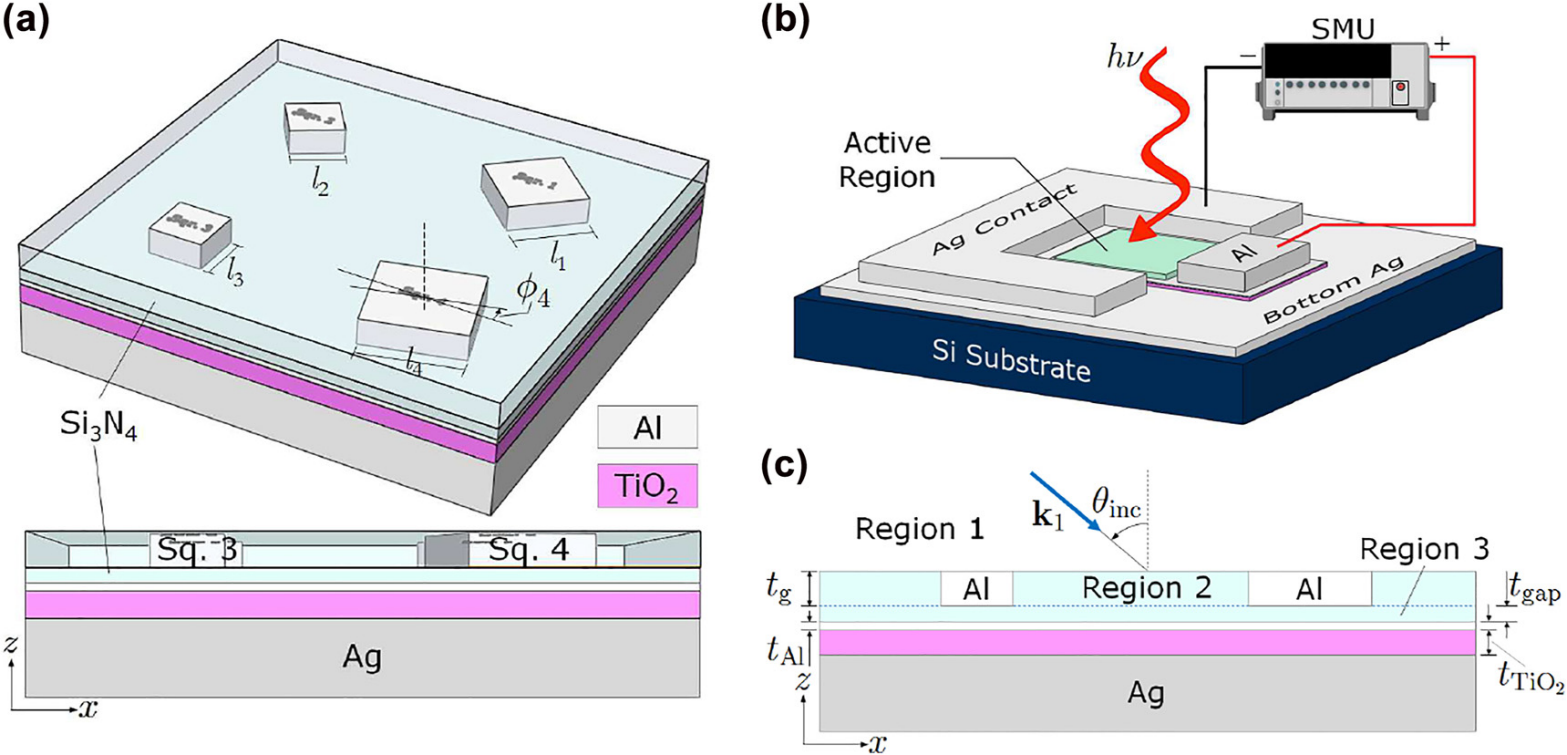
Schematics of the proposed Al-based photonic-plasmonic Schottky photodetector: (a) the structure in one unit cell of the active region and its cross-sectional view, (b) the photodetector showing electrical contacts and the test configuration for current-voltage measurements, and (c) the 2D model (*∂*/*∂y* = 0) for the analytical treatment and modal analysis of the proposed design. A thin Si_3_N_4_ film (region 3) with thickness *t*_gap_ is introduced between the nano-square prisms and planar-Al film to form GPP resonators, enabling the existence of quasi-bound/bound supermodes (Section 6). Region 2 in (c) is the grating region composed of Al strips and Si_3_N_4_. Photon energy conversion is to take place mainly in the planar-Al film (right above the TiO_2_ film).

The proposed hot-electron-based PD is schematically illustrated in Figure 1(a) and (b). The active area is comprised of four Al nano-square prisms of different edge lengths (*l*_*i*_) and rotation angles (*ϕ*_*i*_, measured from the *x* axis) in one unit cell in a metal-dielectric multilayered configuration. Gap plasmon polariton (GPP) resonators are formed by nano-square prisms and the planar Al film with a uniform silicon nitride (Si_3_N_4_) in between. They are periodically arranged to form a two-dimensional (2D) metal grating. Titanium dioxide (TiO_2_) (electron affinity *χ* = 3.9 eV [26]) as an insulator for *λ*_0_ > 430 nm [27] separates the planar-Al film serving as the photon energy absorber from the Ag bottom layer on top of a silicon substrate. The physical reasoning behind the use of Al for photon energy conversion is further detailed in the supplementary material. The periods of nano-prisms in the *x* and *y* directions are set equal so that Λ_*x*_ = Λ_*y*_ = Λ, resulting in a 2D square lattice on the *xy*-plane for polarization-insensitive optical characteristics.

With the introduction of a uniform Si_3_N_4_ gap/film [region 3 in Figure 1(c)] separating the Al-modulated region from the Al–TiO_2_–Ag geometry, how the grating period is determined for maximizing absorption in the planar-Al film may depend on how the space harmonics are required to behave upon leaving the grating region. Consider first a general 2D grating (i.e. a 3D electromagnetic problem). For a forward-diffracted wave of order (*m*, *n*), m,n∈Z , to be evanescent (propagating) in the uniform region contiguous to the grating, the *z*-directed wave number given by

(1) $$k_{3,m,n}\cdot \hat{z}={\left[k_{0}^{2}{\left(n_{3}-j\kappa_{3}\right)}^{2}-\left(\sigma_{x,m}^{2}+\sigma_{y,n}^{2}\right)\right]}^{1/2}$$

must be negative imaginary (positive real), where *σ*_*x*,*m*_ (*σ*_*y*,*n*_) denotes the *x*-directed (*y*-directed) wave vector inside the grating:

(2) $$\sigma_{x,m}=k_{0}\sin\theta_{\text{inc}}\cos\phi_{\text{inc}}-mK_{x},$$

(3) $$\sigma_{y,n}=k_{0}\sin\theta_{\text{inc}}\sin\phi_{\text{inc}}-nK_{y},$$

$\hat{z}$ is the unit vector in the *z* direction, ${\tilde{n}}_{c,3}=n_{3}-j\kappa_{3}$ the complex refractive index in region 3, *θ*_inc_ (*ϕ*_inc_) the incident elevation (azimuthal) angle, and *K*_*x*_ and *K*_*y*_ the grating vectors in the *x* and *y* directions, respectively. In cases where *κ*_3_ = 0 and Λ_*x*_ = Λ_*y*_ = Λ under normal incidence, we obtain

(4) $$\Lambda <\sqrt{m^{2}+n^{2}}\left(\frac{\lambda_{0}}{n_{3}}\right)$$

for a forward-diffracted wave of order (*m*, *n*) to be evanescent in region 3.

Since the square prisms are arranged in a 2D square lattice, there exists a second period of $\sqrt{2}\Lambda$ . Accordingly, two forward-diffracted orders (*m*, *n*) and (*p*, *q*) can satisfy the same evanescent or propagating condition for a Λ if and only if they differ by a factor of $\sqrt{2}$ , that is, $\sqrt{2}\sqrt{m^{2}+n^{2}}=\sqrt{p^{2}+q^{2}}$ . Hence with a proper choice of Λ, two GPP modes or even supermodes sustained by the whole PD may be concurrently excited using two related diffracted orders. Examples of such combinations are: (±1, 0) and (±1, ±1), (±1, ±1) and (±2, 0), (±2, 0) and (±2, ±2), (±2, ±2) and (±4, 0), to name a few. Considering the operating wavelength *λ*_0_ = 638.9 nm and ease of fabrication, the period is limited to within 899 nm (*n*_3_ = *n*_Si_3_N_4__ = 2.0098 [28]), enforcing the (2, 2) order and all orders above it evanescent. As will be elaborated later, concurrent excitation of two or more supermodes using two primary periods (Λ and $\sqrt{2}\Lambda$ ) may significantly enhance the absorptance in the planar-Al film.

## 3 Theoretical formulation

Because of the complexity of the proposed hot-electron-based PD, it is necessary to conduct some fundamental investigations in the framework of a 2D electromagnetic problem. As shall be seen later, the theoretical formulation lays the groundwork for our understanding of the one-to-one correspondence between a specific diffracted order and power absorption in any periodic or uniform layer of interest in this and other similar structures.

The analytical treatment of a 1D metallic grating in a multilayered configuration based on the rigorous transmission-line network approach (without approximations) has been described in relative detail in [21] and will only be summarized briefly here. In this treatment, the fields in the grating region [region 2 in Figure 1(c)] are expanded in terms of the space-harmonic components exp(−*j* ***σ***_*i*_ ⋅ **x**), $j=\sqrt{-1}$ , with *z*-dependent amplitudes that satisfy the transmission-line equations. The Floquet wave vector ***σ***_*i*_, i∈Z , is given by **σ**_*i*_ = **k**_*g*_ − *i* **K**, where **k**_*g*_ denotes the refracted version of the incident wave vector inside the grating and **K** represents the grating vector. Such field expressions together with the complex Fourier series-expanded permittivity of the metallic grating are then substituted into the source-free Maxwell’s curl equations, leading to an infinite set of second-order differential equations for modal currents *I*_*i*_(*z*) in the grating region. In matrix form, we have

(5) $$d^{2}\underline{I^{\prime }}\left(z\right)/dz^{2}=-\underline{\underline{\Omega }}\;\underline{I^{\prime }}\left(z\right),$$

for transverse-magnetic (TM) polarization with the coefficient matrix

(6) $$\underline{\underline{\Omega }}=\underline{\underline{C}}\left\{k_{0}^{2}\underline{\underline{I}}-\underline{\underline{\sigma_{x}}}{\underline{\underline{C}}}^{-1}\underline{\underline{\sigma_{x}}}\right\}.$$

where $\underline{\underline{C}}$ is an *N_t_* × *N_t_* square matrix (after judicious truncation) with elements ${\left(\underline{\underline{C}}\right)}_{ab}=c_{ab}$ , *a* − *b* = *n* being the Fourier coefficients *c*_*n*_’s of the grating profile, *k*_0_ denotes the free-space wave vector and $\underline{\underline{I}}$ the *N_t_* × *N_t_* identity matrix (*N_t_*: the number of space harmonics inside the grating region).

The modal current $\underline{I}\left(z\right)$ and voltage $\underline{V}\left(z\right)$ waves in the grating region are subsequently obtained by applying eigen-decomposition to $\underline{\underline{\Omega }}$ and solving Eq. (5). The results are repeated here [21]:

(7) $$\underline{I^{\prime }}\left(z\right)=\underline{\underline{P}}\left[\exp\left(-j\sqrt{\underline{\underline{D}}}\;z\right){\underline{I^{\prime }}}^{+}-\exp\left(j\sqrt{\underline{\underline{D}}}\;z\right){\underline{I^{\prime }}}^{-}\right],$$

(8) $$\underline{V^{\prime }}\left(z\right)=\underline{\underline{Z_{g}}}\left[\exp\left(-j\sqrt{\underline{\underline{D}}}\;z\right){\underline{I^{\prime }}}^{+}+\exp\left(j\sqrt{\underline{\underline{D}}}\;z\right){\underline{I^{\prime }}}^{-}\right],$$

with

(9) $$\underline{\underline{Z_{g}}}\equiv {\underline{\underline{C}}}^{-1}\underline{\underline{P}}\sqrt{\underline{\underline{D}}}/\left(\omega \epsilon_{0}\right),$$

where $\underline{\underline{D}}$ and $\underline{\underline{P}}$ are matrices composed of the eigenvalues and the corresponding eigenvectors of $\underline{\underline{\Omega }}$ , respectively.

On the other hand, the counterparts of Eqs (7) and (8) in a uniform region obey the conventional transmission line current and voltage equations as follows:

(10) $$\underline{I}\left(z\right)=\underline{\underline{Y_{c}}}\left[\exp\left(-j\underline{\underline{\kappa }}\;z\right){\underline{V}}^{+}-\exp\left(j\underline{\underline{\kappa }}\;z\right){\underline{V}}^{-}\right],$$

(11) $$\underline{V}\left(z\right)=\left[\exp\left(-j\underline{\underline{\kappa }}\;z\right){\underline{V}}^{+}+\exp\left(j\underline{\underline{\kappa }}\;z\right){\underline{V}}^{-}\right],$$

where $\underline{\underline{\kappa }}$ and $\underline{\underline{Y_{c}}}$ are diagonal matrices with entries ${\left(\underline{\underline{\kappa }}\right)}_{ab}=\kappa_{n}\delta_{ab}$ and ${\left(\underline{\underline{Y_{c}}}\right)}_{ab}=Z_{0,n}^{-1}\delta_{ab}$ , where

(12) $$\kappa_{n}=\sqrt{k_{0}^{2}\epsilon_{r}-{\left(\sigma_{n}\cdot \hat{x}\right)}^{2}},$$

(13) $$Z_{0,n}=\kappa_{n}/\left(\omega \epsilon_{0}\epsilon_{r}\right)\;\text{for TM waves},$$

*δ_ab_* is the Kronecker delta symbol, and ${\underline{V}}^{+}$ and ${\underline{V}}^{-}$ are the column vectors representing the respective amplitudes of the forward and backward-propagating modal voltages of all diffracted orders *i*.

Equations (7)–(11) are basic equations required to derive the reflection coefficient, input impedance, and voltage and current transfer matrices associated with any grating and uniform layers. In such a framework, power absorption within any arbitrary layer (uniform or periodic) translates to the difference in total powers delivered to the input and load ends of the associated transmission-line network. The total power is computed in terms of $\underline{V}\left(z^{\prime }\right)$ and $\underline{I}\left(z^{\prime }\right)$ at the input (*z*′ = −*t*) and load/output end (*z*′ = 0) (*z*′: a local coordinate system, *t*: the layer thickness) and summed over all space harmonics inside the grating or diffracted orders outside of the grating.

The formulation is rigorous without approximations and can be applied to any planar structure with arbitrary numbers of grating and uniform layers. It is also worth noting that although the coefficient matrix $\underline{\underline{\Omega }}$ is an infinite matrix, the calculated results are attainable to any arbitrary level of accuracy if it is judiciously truncated in practice.

## 4 General absorptance characteristics with varying gap thicknesses and filling factors

**Figure 2:**
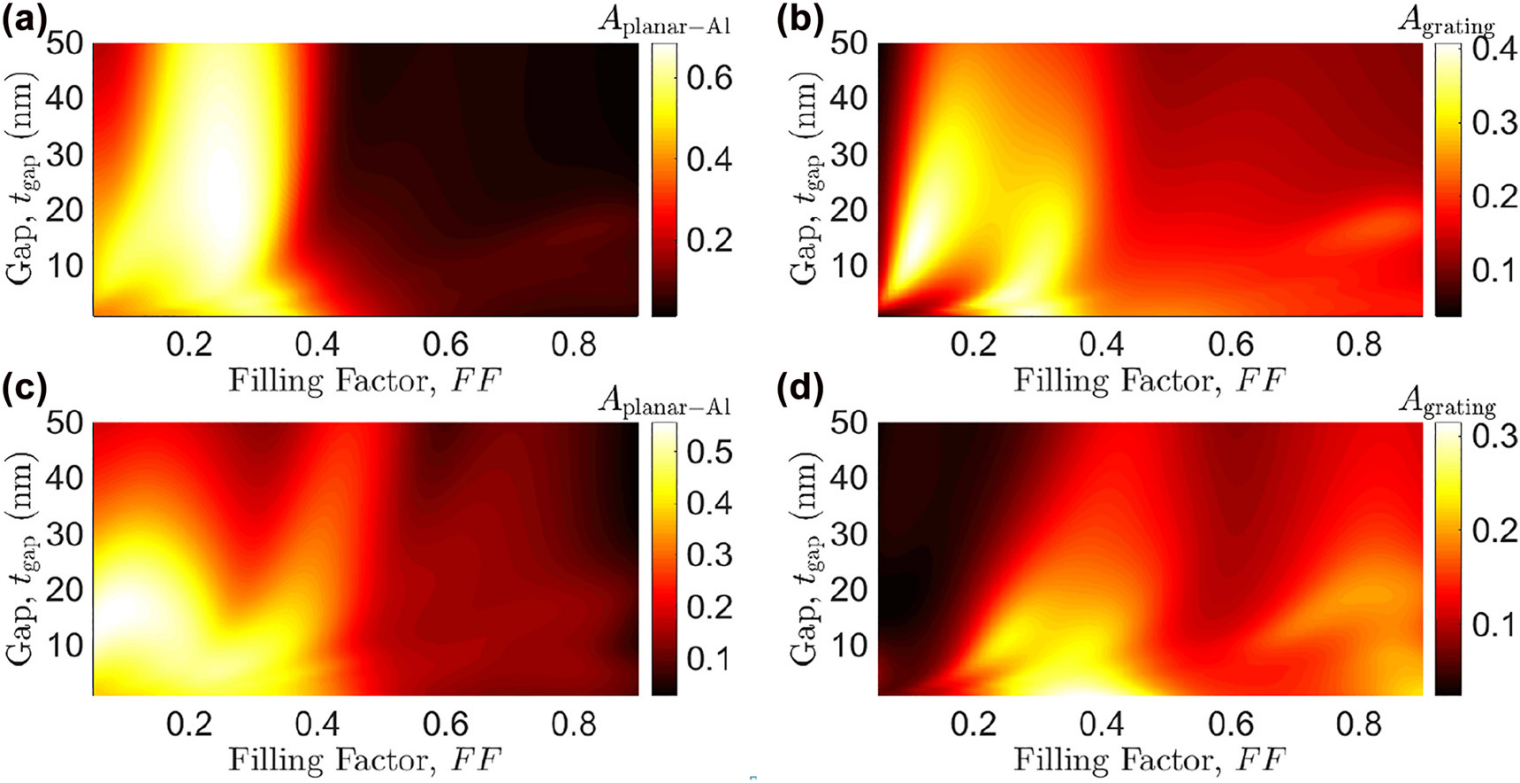
Calculated absorptance dependence of the proposed PD [2D model in Figure 1(c) with only one Al strip] on the filling factor and Si_3_N_4_ gap thickness at *λ*_0_ = 638.9 nm under normal incidence of a TM-polarized plane wave: (a) and (b) are *A*_planar-Al_ and *A*_grating_ for Λ = 600 nm, respectively, while (c) and (d) are their counterparts for Λ = 850 nm. The thickness of the Al grating, planar Al, TiO_2_, and bottom Ag films is 50, 15, 30, and 50 nm, respectively, while that of the Si substrate is 500 μm. The total number of space harmonics *N_t_* retained in the calculations is 201.

Before conducting intensive 3D numerical computations, the absorptance (*A*) characteristics of the proposed PD, *A*_planar-Al_ and *A*_grating_, as a function of gap thickness *t*_gap_ and filling factor *FF* were investigated using the theoretical formulation described above, as shown in Figure 2. The 2D model used in the analysis is given in Figure 1(c) but with only a single Al strip. The filling factor is defined as the fraction of the grating period occupied by the Al strip.

**Figure 3:**
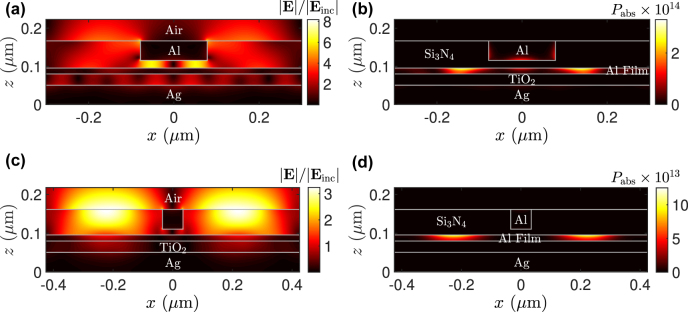
Normalized electric field (magnitude) |**E**|/|**E**_inc_| and power absorption *P*_abs_ (W/m^2^) in a fundamental 1D Al grating in multilayered configuration under normal incidence of a TM wave at *λ*_0_ = 638.9 nm: (a) and (b) are for Λ = 600 nm, whereas (c) and (d) are for Λ = 850 nm, all numerically computed at their corresponding peak *A*_planar-Al_ values shown in Figure 2. The *FF* and *t*_gap_ are 0.26 and 21 nm (0.08 and 16 nm) for Λ = 600 nm (Λ = 850 nm), respectively.

In general, a relatively strong *A*_grating_ manifests the existence of GPPs between the Al strip and planar Al film that leads to a strong *A*_planar-Al_ in the same region on the *FF*–*t*_gap_ plane. Because of the enhanced electric field intensities at the upper and lower interfaces of the Si_3_N_4_ gap, power absorptions within the Al film and Al strip are concurrently increased. This is found true after an extensive study of the field distributions at differing, key combinations of *FF* and *t*_gap_ for differing periods, as shown in Figure 3(a) and (b) as a representative case.

On the contrary, a relatively high *A*_planar-Al_ may also be attained with much weaker GPPs in the present structure. This is illustrated in Figure 3(c) and (d), where strong field strengths between Al strips inside the grating (region) and high absorbed power *P*_abs_ off-strip within the planar Al film are observed. In this particular case with *FF* = 0.08 and *t*_gap_ = 16 nm, absorption in the Al film is mainly contributed by the zeroth diffracted order as the grating is only slightly modulated, resulting in very small diffraction efficiencies of all other diffracted orders. These results reveal the feasibility of enhancing *A*_planar-Al_ directly underneath the Al strips (in 2D) or nano prisms (in 3D) using GPPs with a proper combination of period, gap, and filling factor.

## 5 Spectral and field analyses of hot-electron-based Schottky PD with 2D periodic rotated nano-square prisms

**Figure 4:**
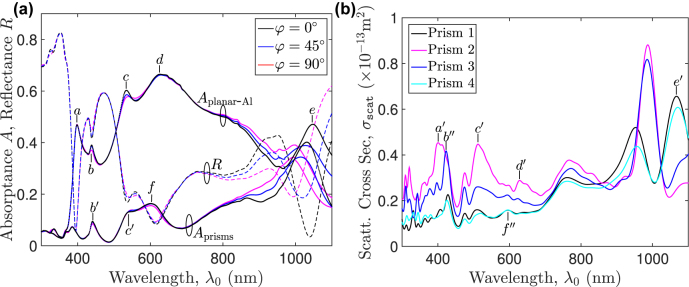
Spectral behaviors of the present Schottky PD [Figure 1(a)] at normal incidence of a plane wave: (a) absorptance *A* and reflectance *R* spectra at differing polarization angles of *φ* = {0°, 45°, 90°} relative to the plane of incidence and (b) scattering cross section *σ*_scat_ of each single nano-square prism positioned at the center of one unit cell computed at *φ* = 0°. Peak wavelengths in (b) close to those of *A*_planar-Al_ in (a) are labeled using the primed/double primed letters in one-to-one correspondence.

Following the studies of absorptance dependence on key parameters of interest, a polarization-insensitive, sub-optimum, broadband Schottky PD is developed (Table 1). Figure 4(a) shows its absorptance and reflectance spectra, exhibiting a peak *A*_planar-Al_ value of >66% at *λ*_0_ = 627 nm and of more than 60% for *λ*_0_ ∈ [588, 693] nm. They remain nearly invariant for wavelengths up to 800 nm as the polarization angle *φ* (measured relative to the plane of incidence or the *xz* plane) changes from 0° (TM or *p* polarization) to 90° (TE or *s* polarization). Further investigations reveal that the 56%-*A*_planar-Al_ bandwidth of the proposed structure is about 41 nm broader than that of the design with zero rotation angles, while the peak absorptance difference between the two is only 0.61%. Also shown in Figure 4(a) is the combined absorptance spectrum of the four rotated square prisms whose peaks closely match those of *A*_planar-Al_. Note that since hot electrons contributing to the photocurrent are almost all generated in the planar-Al film, the absorptance there (i.e. *A*_planar-Al_) is maximized, while that in the nano-square prisms is reduced to its minimum.

**Table 1:** Sub-optimum structure parameters (with consideration of ease of fabrication) obtained based on 3D finite-difference-time-domain computations at *λ*_0_ = 638.9 nm. All lengths are in units of nanometers and angles in degrees.

Λ	*l*_1_	*l*_2_	*l*_3_	*l*_4_	*t*_*g*_	*t*_gap_	*t*_planar-Al_	*t*_TiO_2__
870.34	146.98	100	100	180	47.29	20	10	35
		*ϕ*_1_	*ϕ*_2_	*ϕ*_3_	*ϕ*_4_			
		42.48°	33.80°	0°	16.02°			

To quantify each possible GPP resonance, the scattering cross section *σ*_scat_(*λ*_0_) of each nano square prism in the absence of the other three is investigated. It is defined as

(14) $$\sigma_{\text{scat}}\left(\lambda_{0}\right)=\frac{\oint_{S_{c}}Re{\left.\left[E_{\text{scat}}\left(r,\lambda_{0}\right)\times H_{\text{scat}}^{*}\left(r,\lambda_{0}\right)\right]\right|}_{r\in S}\cdot ds_{c}}{Re\left[E_{\text{inc}}\left(r,\lambda_{0}\right)\times H_{\text{inc}}^{*}\left(r,\lambda_{0}\right)\right]\cdot a_{k}},$$

where **E**_scat_(**r**, *λ*_0_) and **H**_scat_(**r**, *λ*_0_) [**E**_inc_(**r**, *λ*_0_) and **H**_inc_(**r**, *λ*_0_)] denote the scattered (incident) electric and magnetic field intensities, respectively, * represents the complex conjugate, **a**_*k*_ the unit vector in the direction of wave vector **k**, and *S*_*c*_ the closed surface (enclosing the nano prism) on which the net outward time-average power flow is numerically computed. For easy comparisons, peak wavelengths in Figure 4(b) close to those of *A*_planar-Al_ in Figure 4(a) are labeled using the primed/double primed letters in one-to-one correspondence. The occurrence of GPP resonance at each major *σ*_scatt_ peak is further verified by field distributions across the *xz* plane. It is worth mentioning that in arriving at Figure 4(b), interactions among nano prisms have been ignored due to the constraint of the total field-scattered field formulation. It is apparent that GPP resonance enhances the peaks of *A*_planar-Al_, in particular for *λ*_0_ ∈ [512, 800] nm within which *A*_planar-Al_ ≥ 50%, and further broadens the absorptance bandwidth of the planar-Al film.

**Figure 5:**
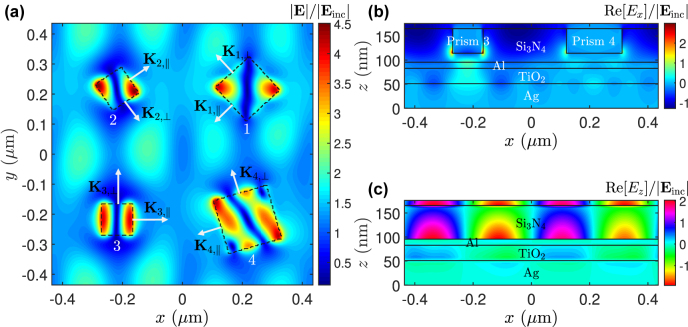
Normalized electric field distribution (normalized to the incident |**E**_inc_| field) across some planes of interest of the proposed PD at *λ*_0_ = 627 nm under normal incidence of an *x*-polarized (*φ* = 0°) plane wave: (a) |**E**| field across the mid-gap with grating vectors **K**_‖_ and **K**_⊥_ associated with each square prism, (b) *E*_*x*_ field (real part) across the *xz* plane passing the centers of square prisms 3 and 4, exhibiting strong coupling of GPPs and SPPs, and (c) *E*_*z*_ field (real part) on the *y* = 0 plane at zero phase angle, showing quasi-surface plasmon resonance at Al-Si_3_N_4_ interface. The orientation of each square prism satisfies Eq. (15), resulting in quasi-surface plasmon resonance at the Si_3_N_4_–Al film interface in (c).

Figure 5 shows the normalized electric field at the peak absorptance wavelength across some planes of interest, revealing GPPs and quasi-surface plasmon resonance within the proposed PD. Strong field magnitudes up to more than four times larger than the incident electric field in the Si_3_N_4_ gaps are clearly seen [Figure 5(a)]. We also observe, at some particular phase angles, strong coupling between the tangential **E** field components of GPPs and SPPs existing at the TiO_2_–Ag interface, as shown in Figure 5(b) as a representative case. Moreover, relatively weak coupling is also noticed between GPPs and the localized SPPs (LSPPs) around the square prisms as *E*_*x*_ and *E*_*y*_ of GPPs are considerably weaker than the *E*_*z*_ field but they are the dominant components of LSPPs.

In the meantime, quasi-surface plasmon resonance at the Si_3_N_4_-planar-Al interface [Figure 5(c)] is also observed, thus further enhancing the absorptance in the preferred planar-Al film. This resonance is produced by the components of grating vectors parallel to the incident polarization and nearly equal in magnitude and point in opposite directions. For instance, as depicted in Figure 5(a), the grating vector **K**_4,‖_ associated with the left sidewall of prism 4 has its *x* component *K*_4,*x*_ = |**K**_4,‖_| cos(16.02°) nearly equal to **K**_3,‖_ in magnitude and points in the −*x* direction, which is just the opposite of **K**_3,‖_. The same argument also applies to the grating vectors associated with square prisms 1 and 2; that is, |**K**_2,‖_| cos *ϕ*_2_ + |**K**_2,⊥_| sin *ϕ*_2_ ≈ |**K**_1,‖_| cos *ϕ*_1_ + |**K**_1,⊥_| sin *ϕ*_1_. In fact, when all prisms are considered, the following expression still holds:

(15) $$\begin{array}{ll} & \underset{i=2,3}{\sum }|K_{i,\|}|\cos\phi_{i}+|K_{i,⊥}|\sin\phi_{i}\\ & \;\approx \underset{i=1,4}{\sum }|K_{i,\|}|\cos\phi_{i}+|K_{i,⊥}|\sin\phi_{i}, \end{array}$$

where **K**_*i*,‖_ (**K**_*i*,⊥_) represents the grating vector parallel (normal) to the *x* axis when prism *i* is at zero rotation angle (i.e. *ϕ*_*i*_ = 0°). However, since the wavenumbers on both sides of Eq. (15) are only nearly identical and stronger field coupling does exist among rotated square prisms, the *E*_*z*_ field oscillations are not perfectly standing still. Thus the square prisms are rotated in such a way to produce the maximum *A*_planar-Al_ via the generation of quasi-surface plasmon resonance at the top boundary of the planar-Al film.

Referring to Figure 5(a), a few points regarding the field distribution in the gap region are worth noting. The field distribution underneath the nano-square prisms is the modal field associated with (leaky) GPP resonance. It depends on the size of the prism together with the symmetry relation between the incident polarization and the orientation of each prism. Note that a square has four lines of symmetry. The connections between the incident polarization and field distributions underneath square prisms are summarized below:

1. For a given square prism, when the incident polarization is parallel to its line of symmetry (on the *xy* plane), a modal field pattern symmetric to that line of symmetry is produced.
2. Strong field distributions completely parallel to the side walls (in the vicinity of opposite vertices) of a prism occur only when the incident polarization is parallel to a nondiagonal (diagonal) line of symmetry.
3. Depending on how the incident polarization is oriented relative to a square prism’s lines of symmetry, the strong field distributions can change from being completely parallel to the side walls to being near the vertices of a prism.

Further information, including field distributions at polarization angles of 45° and 90° and their associated explanations, is provided in the supplementary materials.

## 6 Diffracted-order-resolved analysis of physical processes producing strong absorptance

In the following, we will concentrate on unraveling the relationships between a diffracted order, its associated absorptance in the planar Al film, and the quasibound/bound modal fields sustained in the whole structure. In this regard, while the 2D model shown in Figure 1(c) is analytically analyzed using the theoretical formulation presented in Section 3, the results may provide in-depth physical insights into the 3D structure.

**Figure 6:**
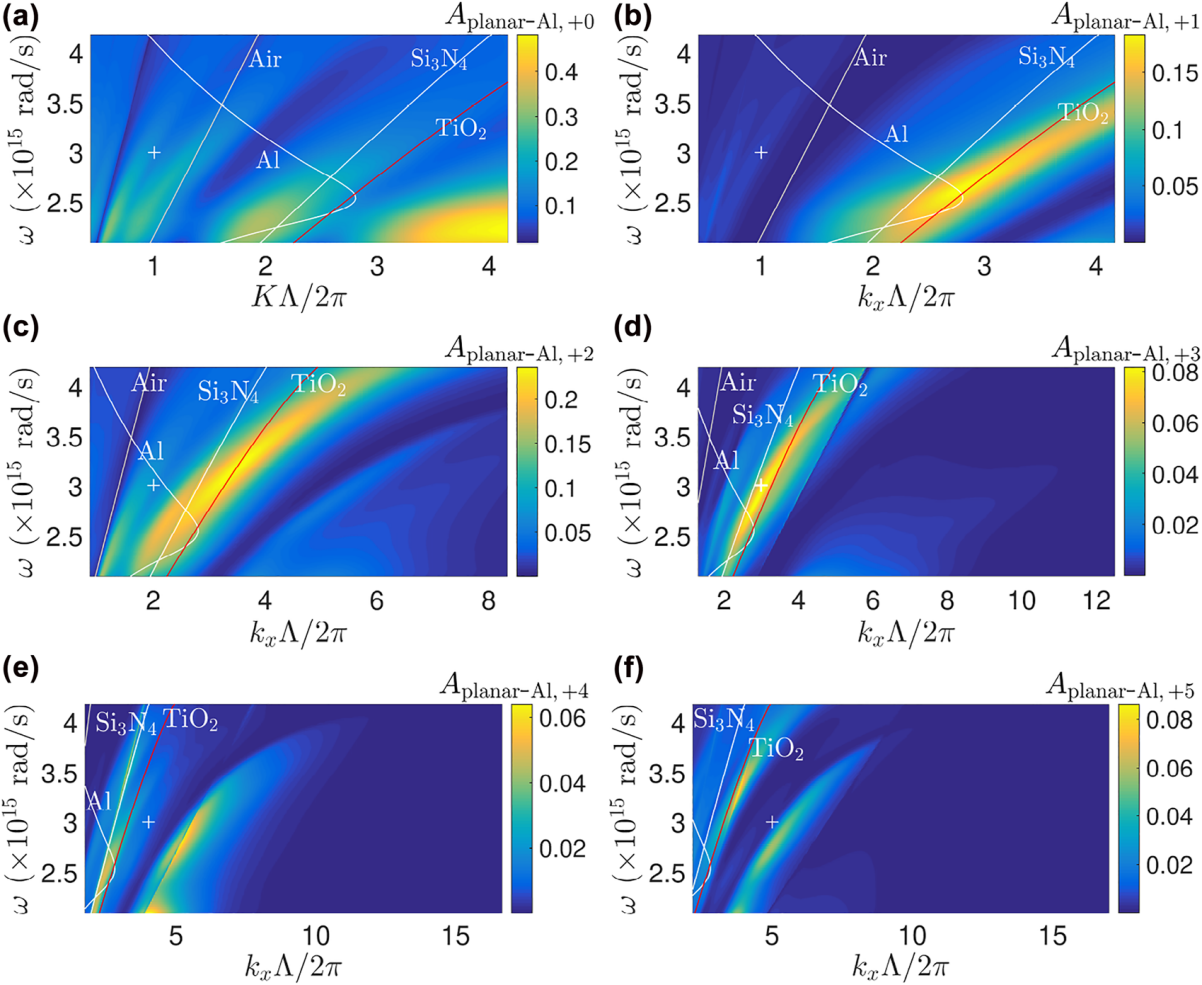
Contribution from the zeroth and the first five positive diffracted orders to *A*_planar-Al_ as a function of angular frequency and normalized grating vector (for *i* = 0) or normalized parallel wave vector (for *i* ≥ 1): (a)–(f) correspond to the zeroth (*i* = 0), first (*i* = +1), second (*i* = +2), third (*i* = +3), fourth (*i* = +4), and the fifth (*i* = +5) diffracted orders, respectively. The light lines of Al, air, Si_3_N_4_, and TiO_2_ are also presented. The point corresponding to the sub-optimum design operating at *λ*_0_ = 627 nm is marked by a cross symbol in each sub-figure. In (b)–(f), *k*_*x*_ = *i*2*π*/Λ, *i* = {1, 2, 3, 4, 5} due to normal incidence.

Figure 6 shows the individual contribution from the first six diffracted orders to *A*_planar-Al_ as a function of angular frequency *ω* and normalized grating vector (for *i* = 0) or normalized parallel wave vector (for *i* ≥ 1). The 2D model is a *y*-cut through prisms 3 and 4 [Figure 1(c)] whose effective strip widths in the *x* direction may be taken as $w_{\text{eff},i}=\sqrt{2}l_{i}cos\left(45{}^{\circ}-\phi_{i}\right)\pm \delta w_{i}$ , where *δw* is a small correction term to include the fringing effect along the edges of a prism. To generalize the computational results to other wavelengths, the filling factors obtained at Λ = 870.34 nm remain unchanged as *k*_*x*_ (or equivalently 1/Λ) varies. The absorptance in the Al film associated with the *i*th order *A*_planar-Al,*i*_ is subsequently obtained. Notice that *A*_planar-Al,+*i*_ = *A*_planar-Al,−*i*_ for the same *i* value due to normal incidence.

As seen in Figure 6(a), the absorptance of the zeroth diffracted order of the present work (marked by a cross symbol) lies within the air light cone, an unbound mode region in the dispersion diagram. At normal incidence, photoexcited electrons due to photon energy absorption associated with the zeroth order wave involve no plasmonic energy conversion. Meanwhile, although the *i* ≥ 4 diffracted orders [Figure 6(e) and (f)] correspond to truly bound modes outside the TiO_2_ light cone, their associated *A*_planar-Al,+*i*_’s are relatively small when compared to those of the second and third orders. In contrast to the bound modes, the third order wave [Figure 6(d)] corresponds to quasibound, leaky mode as it situates between the light lines of Si_3_N_4_ and TiO_2_, suggesting partial power leakage into the high-index TiO_2_ film. Referring to Figure 5(b), such leakage or power transfer may be caused by the coupling of tangential field components between Si_3_N_4_–Al and TiO_2_–Ag heterojunctions, thus increasing the total absorptance in the Al film.

**Figure 7:**
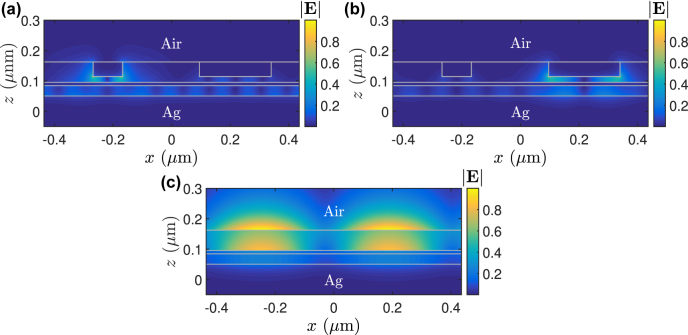
Modal electric field (magnitude) of the eigenmode sustained in the 2D structure with (a) Re[*N*_eff_] = 2.192513, a quasibound supermode excited by the *i* = ±3 diffracted orders and (b) Re[*N*_eff_] = 2.864253, a bound supermode excited by the *i* = ±4 order (see Table 2). The field shown in (c) is the leaky SP mode with Re[*N*_eff_] = 1.453872 of the same 2D structure as in (a) and (b) but deprived of the Al strips.

To understand how each diffracted order contributes to *A*_planar-Al_, the supermodes (i.e. eigenmodes) sustained by the 2D structure [Figure 1(c)] are numerically computed. Periodic and metal boundary conditions are respectively specified in the *x* and *z* directions with the latter being positioned, following convergence test results, 650 nm (100 nm) away from the top (bottom) surface of the 2D structure. Effective indexes (real part) of the supermodes Re[*N*_eff_], the normalized parallel wave vectors |*i*|*λ*_0_/Λ, *A*_planar-Al,*i*_, and the diffraction efficiency *DE*_*i*_ associated with the *i*th diffracted order calculated in region 3 are listed in Table 2. Close agreement is observed between Re[*N*_eff_] and the normalized *k*_*x*_ for *i* = {±3, ±4}. The modal electric fields of the supermodes corresponding to *i* = ±3 and *i* = ±4 orders are shown in Figure 7(a) and (b), each of which exhibits relatively strong field strength in one of the gap regions and the planar Al–TiO_2_–Ag structure. As pointed out earlier, the former is inherently a leaky mode with partial power leakage/transfer into the higher-index TiO_2_ film. Thus this supermode may be treated as an establishment of coupled leaky GPP and bound SPP modes, causing significantly higher *A*_planar-Al,±3_.

**Table 2:** Comparison of normalized parallel wave vectors *k*_*x*_/*k*_0_ associated with the first six diffracted orders and modal indexes of the supermodes sustained by a 2D slice through the centers of square prisms 3 and 4 at *λ*_0_ = 627 nm. The corresponding effective strip widths are 100 and 242.77 nm, respectively.

Along *x* axis; Λ = 870.34 nm				
Modal index, Re[*N*_eff_]	Normalized *k*_*x*_, |*i*|*λ*_0_/Λ	Diffracted order, *i*	*A*_planar–Al,*i*_ (%)	*DE*_*i*_ (%)
–	0	0	14.08	14.41
–	0.720408	±1	1.226	1.234
–	1.440816	±2	7.175	7.238
2.192513	2.161219	±3	7.098	7.209
2.864253	2.881626	±4	1.037	1.088
–	3.602032	±5	0.217	0.452

In contrast to the third and fourth orders, we found the second diffracted order could excite a leaky SP mode in the *planar* Si_3_N_4_–Al–TiO_2_–Ag multilayered structure in the absence of the Al-modulated region. Figure 7(c) depicts the modal electric field of the eigenmode sustained in the same 2D model described above but is deprived of the Al strips. It exhibits leakage radiation into the air and its effective index is fairly close to the normalized *k*_*x*_ at *λ*_0_ = 627 nn. As apparent, one period of 870.34 nm can accommodate approximately two guided wavelengths *λ*_*g*_’s at *λ*_0_ = 627 nm. Consequently, we see that the quasi-standing-wave pattern shown in Figure 5(c) may stem from the second diffracted order that excites this leaky surface mode since the separation between field maxima is *λ*_g_/2 (i.e. four field peaks in one period).

The quasibound supermode that constitutes a significant portion of absorption in the Al film is also found in the 2D slice along the cell diagonal, as given in Table 3. In the actual 3D structure, the period along the cell diagonal $\left(\sqrt{2}\Lambda \right)$ is the second shortest for all prisms and is “seen” by diffracted orders (±*i*, ±*i*). In this case, the effective Al strip width is taken as $w_{\text{eff},i}^{\prime }=\sqrt{2}l_{i}cos\left(\phi_{i}\right)+\delta w_{i}^{\prime }$ . Close agreement is apparent between the effective index of the quasibound (bound) supermode and the normalized *k*_*x*_’s of the *i* = ±4 (*i* = ±5) orders. Also, we see that *A*_planar-Al, 0_ for $\Lambda =870\times \sqrt{2}$ nm is very close to its counterpart for Λ = 870 nm, while the quasibound supermode excited by *i* = ±4 orders contributes the most to the total absorptance in the Al film. Further, though the bound supermode with Re[*N*_eff_] = 2.546450 may be excited by the fifth order, their associated *A*_planar-Al,±5_ is very small because of the small diffraction efficiency (3.642 × 10^−2^%).

**Table 3:** Comparison of normalized parallel wave vectors *k*_*x*_/*k*_0_ associated with the first six diffracted orders and modal indexes of the supermodes sustained by the 2D structure sliced along the cell diagonal passing prisms 1 and 3 at *λ*_0_ = 627 nm. The corresponding effective strip widths are 156.42 and 164.31 nm, respectively.

Along the cell diagonal; $\Lambda =870.34\times \sqrt{2}$ nm				
Modal index, Re[*N*_eff_]	Normalized *k*_*x*_, |*i*|*λ*_0_/Λ	Diffracted order, *i*	*A*_planar–Al,*i*_ (%)	*DE*_*i*_ (%)
–	0	0	13.89	13.97
–	0.509405	±1	1.925 × 10^−2^	1.937 × 10^−2^
–	1.018811	±2	8.526 × 10^−2^	8.587 × 10^−2^
–	1.528216	±3	2.315 × 10^−2^	2.337 × 10^−2^
2.022752	2.037622	±4	15.76	15.98
2.546450	2.547027	±5	3.550 × 10^−2^	3.642 × 10^−2^

It is noted that from Tables 2 and 3 and Eq. (4), the quasibound and bound eigenmodes sustained by the 2D structure may be only excited by diffracted waves that are evanescent in the uniform region contiguous to the Al grating [i.e. region 3 in Figure 1(c)]. For a 1D grating (i.e. a 2D problem), Eq. (4) reduces to Λ < |*i*|*λ*_0_/*n*_3_, giving rise to the period below which the second, third, and the fourth diffracted orders is evanescent are 623.51, 935.26, and 1247.02 nm, respectively (*λ*_0_ = 627.0 nm, *n*_3_ = *n*_Si_3_N_4__ = 2.0112). Thus with Λ = 870.34 nm (and $\Lambda =870.34\times \sqrt{2}=1230.85$ nm along the cell diagonal), all quasibound and bound eigenmodes are excited by |*i*| ≥ 3 (|*i*| ≥ 4) orders that are all evanescent in region 3.

## 7 Experimental demonstrations and discussions

**Figure 8:**
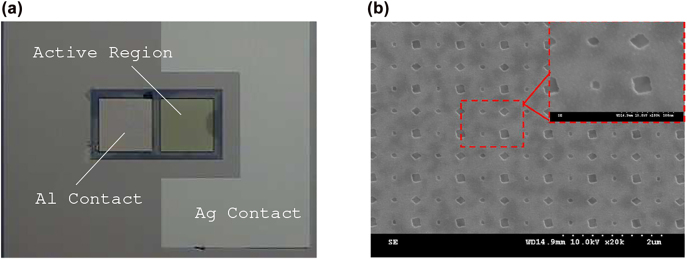
The fabricated photonic-plasmonic Schottky PD: (a) the optical image (at 500×), showing the layout of top-Al and bottom-Ag electrodes along with the active region having an area of $∼4.6457\times 10^{-2}$ mm^2^ and (b) the scanning electron microscope image of the fabricated array of nano-square prisms. The optical image was taken after the deposition of Si_3_N_4_ film.

The proposed Al-based PD was fabricated (see Figure 8) and experimentally demonstrated. The active region with periodically-arranged nano-square prisms on top of it is about 4.6457 × 10^−2^ mm^2^ in area, whereas the thickness of nano-square prisms, Si_3_N_4_ gap, planar Al, TiO_2_, and the bottom Ag films is approximately 47, 15, 12, 35, and 50 nm, respectively. The TiO_2_ film was grown by atomic layer deposition at 100 °C using TiCl_4_ as the precursor. To bring the temperature of the sample to its minimum during the electron-beam (e-beam) evaporation of the metals in the active region, the deposition rate was controlled at approximately 0.1 $\overset{{}^{\circ}}{A}/s$ under the base pressure of 4 × 10^−6^ torr or smaller. Nano-square prisms were fabricated using standard e-beam lithography, anisotropic etching of the Si_3_N_4_ film, e-beam evaporation of Al, and lift-off techniques.

**Figure 9:**
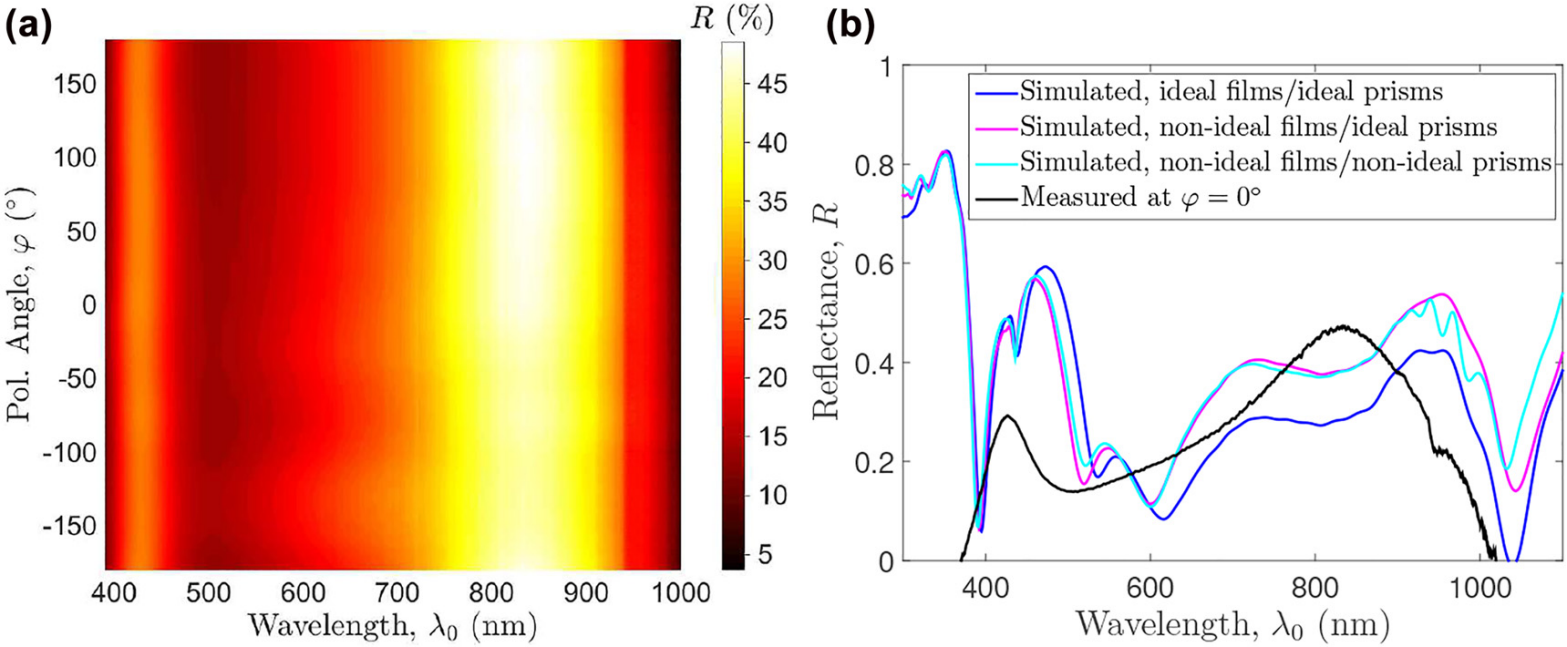
Optical characterization of the fabricated photodetector and comparison with numerical results: (a) the measured reflectance *R* spectrum within a full range of polarization angle *φ*. (b) comparisons of the measured and simulated *R* spectra, where simulation results of three representative structures are presented: 1. The ideal case described in Table 1, 2) non-ideal film thicknesses (*t*_planar-Al_, *t*_gap_) = (12, 15) nm but ideal square prisms, and 3) non-ideal film thicknesses (*t*_planar-Al_, *t*_gap_) = (12, 15) nm and non-ideal nano-square prisms 1, 2, and 3.

Figure 9(a) shows the measured reflectance spectrum (normalized to that of a high-reflectance silver mirror) as a function of polarization angle *φ*. The test configuration is identical to that described in [21]. It has to be noted that as the rotation angle and the *xy* dimensions of each nano-square prism differ from one another, there exists no rotational symmetry and a full revolution of the device about the *z* axis is required to fully characterize the polarization dependence of the reflectance spectrum. The measured reflectance is to a large extent polarization insensitive and is constantly <23% for *λ*_0_ ≈ [449.9, 648.3] nm across the angular spectrum.

In consideration of assessing how the non-ideal square prisms seen in the fabricated PD, particularly prisms 1, 2, and 3, affect its optical characteristics, comparisons of the measured reflectance spectrum and simulated results of three representative structures are made, as shown in Figure 9(b). In cases where non-ideal square prisms are considered in numerical models, diamond prisms having similar dimensions and the same rotation angles as their counterparts are used to approximate square prisms 1, 2, and 3. On the other hand, non-ideal film thicknesses are considered only for the planar-Al film and Si_3_N_4_-gap as they are the two most critical regions in the PD. All other structural parameters are assumed identical to those given in Table 1. As apparent, simulation results suggest that the combination of film thicknesses, including the 2D metal grating composed of nano-square prisms, dictates the spectral behaviors of the device. Non-square-shaped prisms seen in the fabricated PD may thus have a relatively weak impact on the overall device performance for *λ*_0_ < 900 nm.

**Figure 10:**
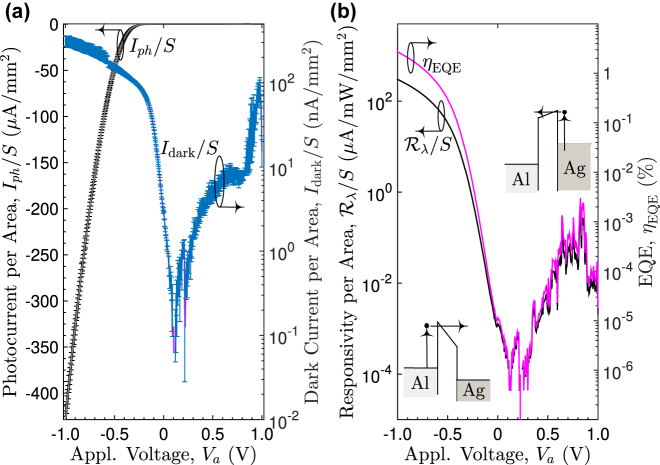
Dependence of the measured dark current and photocurrent per unit area (in units of nA/mm^2^ and μA/mm^2^, respectively) on the applied bias with error bars of one standard deviation (a) and the corresponding per-unit-area responsivity (μA/mW/mm^2^) and EQE (b) of the fabricated PD under a constant light illumination of 1.421 mW at *λ*_0_ = 638.9 nm.

The photoresponse of the fabricated device at *λ*_0_ = 638.9 nm as a function of bias voltage *V*_*a*_ conducted within a shielding box at room temperature is shown in Figure 10. Also shown is the dark current measured in the same controlled environment. The device was configured with the planar-Al (bottom Ag) film connected to the positive (negative) terminal of a Keithley 6430 source meter. Each curve in Figure 10(a) is the average of five measured *I*–*V* curves with each data point being the mean of 10 measurement readings. The dark current is in the sub-nano ampere (nA) order for *V*_*a*_ ∈ [−0.09, 0.875] V and is less than 14.5 nA at *V*_*a*_ = −0.9951 V. When under constant light illumination, the PD exhibits a rectifying characteristic typical of Schottky junction. The photocurrent increases as the reverse bias is increased because of the increasing difference in the quasi-Fermi level between both sides of the TiO_2_ film. This leads to a steeper downward slope of the TiO_2_ band edge toward the Ag side [see the inset of Figure 10(b)], resulting in a narrower effective barrier thickness which in turn increases tunneling probabilities of photoexcited electrons generated in the Al film.

Under light illumination of a constant power of 1.421 mW at *λ*_0_ = 638.9 nm, the per-unit-area responsivity $R_{\lambda }/S$ in μA/mW/mm^2^ (responsivity $R_{\lambda }$ in μA/mW) is 0.0110 (5.10 × 10^−4^), 1.9741 (0.0917), 33.3202 (1.5479), 139.8147 (5.2815), and 298.1444 (13.8509) at a voltage bias of −0.1, −0.3, −0.5, −0.7, and −0.9951 V, respectively, while the corresponding external quantum efficiency (EQE) defined as the ratio of the number of electrons in the photocurrent to the number of incident photons is 9.8962 × 10^−4^%, 0.0178%, 0.3004%, 1.0249%, and 2.6878%. Despite a rather thick 35-nm TiO_2_ film, which is seven times thicker than that in [21], the per-unit-area responsivity of the present work is a few times to several orders of magnitude greater than those reported to date based on hot electron processes operating at similar wavelengths and biases [3, 15, 16, 21]. The uniform Si_3_N_4_ gap separating Al nano prisms from the Al film not only enables quasibound/bound-supermode-enhanced absorption but also limits the spatial degrees of freedom during electron transport, thus increasing the average reaching-emission probabilities of hot electrons. The responsivity and EQE can be significantly increased by increasing the active area up to 1 mm^2^.

Comparisons of the present work and recently reported hot-electron-based PDs operating in the visible regime are given in Table 4. The detectivity D* was calculated using the equation [29].

(16) $$D^{*}=\sqrt{S}R_{\lambda }/\sqrt{2eI_{\text{dark}}},$$

where *S*, *e*, and *I*_dark_ denote the active area, elementary charge, and the dark current, respectively. The per-unit-area responsivity of the PD presented in this work is substantially greater than those previously reported. Despite a much smaller active area of our PD, the detectivity achieves 4.3809 × 10^9^ cm Hz^1/2^/W at *λ*_0_ = 638.9 nm. It is worth mentioning that a smaller active area, though may generally have a better high-speed performance due to a smaller *RC* time constant, would produce a smaller photocurrent, resulting in a smaller responsivity and detectivity.

**Table 4:** Comparisons of recently reported hot-electron-based PDs operating in visible frequencies (*S*: active area, *V*_*a*_: applied voltage, $R_{\lambda }$ : responsivity, *λ*_0_: free-space wavelength, $R_{\lambda }/S$ : per-unit-area responsivity, *D* ^*^: detectivity).

Figure of Merits						
Ref.	*S* (mm^2^)	*V*_*a*_ (V)	$R_{\lambda }$ (mA/W)@*λ*_0_	EQE (%)	$R_{\lambda }/S$ (mA/W/mm^2^)	*D* ^*^ (cm Hz^1/2^/W)
[3]	1	0.6	2.4 × 10^−5^@633 nm	–	2.4 × 10^−5^	–
[11]	28	0.3	–	0.2^a^	–	–
[13]	9	−4	70@640 nm^b^	12	7.7778	–
[15]	–	−0.15	0.375 × 10^−3^@450 nm	–	–	–
[16]	126.56	−1	0.36@640 nm	–	2.8445 × 10^−3^	–
[18]	28	0	3.3@450 nm	0.91	0.1179	9.8 × 10^10^
This work	0.046457	−0.9951	13.8509@638.9 nm	2.6878	298.1444	4.3809 × 10^9^

^a^Measured at *λ*_0_ = 530 nm. ^b^In [13], $R_{\lambda }\approx 0.45$ mA/W at a reverse bias of 1 V.

The temporal response of the fabricated PD is quantified by calculating the two-conductor capacitance and estimating the series resistance *R*_*s*_ from the measured log(|*I*|)–*V* curves (dark current and photocurrent) characterized by a distinct linear relationship. Since the electric bias is applied to the planar-Al and bottom Ag films separated by the TiO_2_ film, the capacitance is approximated by the parallel-plate capacitance *C* = *ϵ*_TiO_2__ *S*/*t*_TiO_2__ and is found to be 6.70964 × 10^−11^ F. Since it is well-known that the capacitance is proportional to the surface area of the conductor, in general, the larger the active area, the larger the capacitance, and the worse the rise/fall times would be.

**Figure 11:**
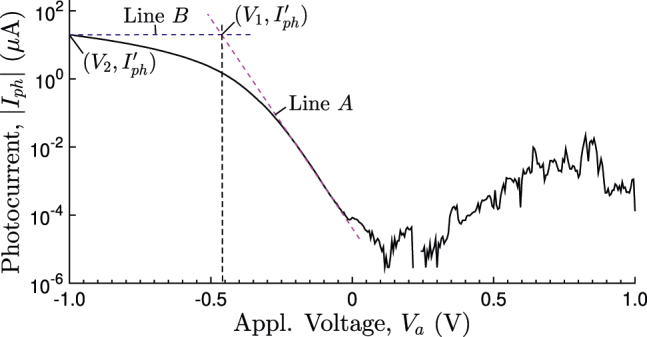
Procedure for estimating the series resistance *R*_*s*_ from the measured log(|*I*|)–*V* curve under light illumination.

On the other hand, the series resistance *R*_*s*_ may be estimated as follows. As shown in Figure 11, with the increase in the reverse bias, the log(|*I*|)–*V* curve deviates from the linear relationship owing primarily to *R*_*s*_. The procedure for estimating *R*_*s*_ from the measured log(|*I*|)–*V* curve is as follows: (1) Draw a tangent line (line *A*) to the linear section of the curve. (2) Draw a horizontal line (line *B*) at a given current value $I_{ph}^{\prime }$ , that deviates from the linear log(|*I*|)–*V* relationship. (3) Two voltage values *V*_1_ and *V*_2_ are obtained; one is at the intersection of lines *A* and *B* [i.e. at $\left(V_{1},I_{ph}^{\prime }\right)$ ], and the other is taken at the point where line *B* intersects the log(|*I*|)–*V* curve [i.e. at $\left(V_{2},I_{ph}^{\prime }\right)$ ]. (4) Determine *R*_*s*_ using the formula $R_{s}=\left(V_{1}-V_{2}\right)/I_{ph}^{\prime }$ . The series resistance in the dark mode is thus estimated to be 55.024 MΩ. The number decreases to about 24.927 kΩ under light illumination, resulting in an *RC* time constant of approximately 1.673 μs. It is worth mentioning that in our device the estimated *R*_*s*_ is nearly independent of the choice of $I_{ph}^{\prime }$ , provided line *B* intersects the log(|*I*|)–*V* curve at some point away from line *A*.

## 8 Conclusions

Hot-electron-based photodetection exploiting pure photoexcitation in a thin Al film and leaky plasmonic modes inclusive of quasibound supermodes and leaky SP mode resonance has been proposed, theoretically investigated, and experimentally demonstrated at *λ*_0_ = 638.9 nm. The innovative design with four nano-square prisms in one unit cell on top of a planar dielectric-metal multilayered structure is shown to be broadband and polarization-insensitive with a peak absorptance in the planar Al film (*A*_planar-Al_) larger than 66%. Using an analytical treatment based on the rigorous transmission-line network formalism and numerical computations of eigenmodes, we see that leaky SP mode resonance with leakage radiation into the air and quasibound supermodes with power leakage via GPP and bound SPP modes coupled between two different heterojunctions may significantly contribute to *A*_planar-Al_. While the leaky SP mode resonance is shown to be produced by a lower propagating diffracted order, quasibound, and bound supermodes can only be launched by higher diffracted orders that are evanescent in the uniform Si_3_N_4_ gap region right above the planar Al film. Further, leaky SP mode resonance occurs if differing grating vectors associated with sidewalls of adjacent square prisms have components pointing in opposite directions and are (nearly) equal in magnitude when summed up vectorially.

The fabricated device is experimentally shown to be polarization insensitive. Comparisons of the measured reflectance spectrum and simulated results of representative structures suggest the control of the combination of layer thicknesses may be more critical than the exact shape of periodically-arranged Al nanostructures. The per-unit-area responsivity, EQE, and the detectivity at *V*_*a*_ = −0.9951 V and *λ*_0_ = 638.9 nm are 298.1444 μA/mW/mm^2^, 2.6878%, and 4.3809 × 10^9^ cm Hz^1/2^/W, respectively. The series resistance obtained from the measured log(|*I*|)–*V* curve gives an *RC* time constant estimate of 1.673 μs under light illumination. The performance is among the best of those previously reported operating at similar wavelengths and biases. Our approach may provide an alternative route and push the research in harvesting hot carriers in metals, which has been considered extremely difficult, one step further toward its practical applications.

## Supplementary Material

Supplementary Material Details
