# Supplementary material for: Photonic-plasmonic hot-electron-based photodetection with diffracted-order-resolved leaky plasmonic mechanisms
Source: Nanophotonics. 2022 Aug 19;11(19):4439–53. doi: 10.1515/nanoph-2022-0370 (PMC11501917; doi:10.1515/nanoph-2022-0370)
Supplement: Supplementary file 1 — Supplementary Material Details [file j_nanoph-2022-0370_suppl.pdf]

Supplementary Material

Yin-Jung Chang*, Ko-Han Shih, and Chun-Yu Hsiao

Photonic-plasmonic hot-electron-based photodetection with diffracted-order-resolved leaky plasmonic mechanisms

https://doi.org/10.1515/sample-YYYY-XXXX

1 Physical reasoning behind using aluminum for photon energy conversion

The physical reasoning behind the use of aluminum (Al) as photon energy absorption material has its root in the electron energy band structure that subsequently determines the joint density of states (JDOS) $\mathcal{J}(h\nu)$ and the energy distribution of joint density of states (EDJDOS) $\mathcal{D}(E_i, h\nu)$ ($h\nu$: photon energy). The JDOS is the density of state pairs (initial and final energy states) involved in the optical process and is written, in the direct (vertical) transition approximation, as

$$\mathcal{J}_{\text{direct}}(h\nu) = \frac{1}{4\pi^3} \sum_{i,f} \int_{\text{FBZ}} f_F(E_i)[1 - f_F(E_f)]\delta\left(E_{f,\mathbf{k}} - E_{i,\mathbf{k}} - h\nu\right) d^3\mathbf{k}, \tag{1}$$

where $f_F(E)$ is the Fermi-Dirac probability function at energy E, $\delta()$ is the Dirac-delta function, and $E_{i,\mathbf{k}}$ and $E_{f,\mathbf{k}}$ denote the initial and final energy states, respectively, at a given $\mathbf{k}$ point (wavevector) in the first Brillouin zone (FBZ). Thus the physical meaning of $\mathcal{J}_{\text{direct}}(h\nu)dh\nu$ is interpreted as the per-unit-volume, occupancy-probability-weighted total number of allowed vertical transitions due to photon energies within the energy interval $[h\nu,\, h\nu + dh\nu]$.

Figure S1 shows the JDOS in one primitive cell ($\mathcal{J}_{\text{direct}} \cdot \Omega_c$) as a function of photon energy $h\nu$ for Al, copper (Cu), and silver (Ag) computed using the actual energy band structures in the framework of direct (vertical) transition. Because of its free-electron-like energy band structure in the vicinity of the W point in the FBZ, Al exhibits no cut-off photon energy below which its JDOS vanishes, leading to the highest JDOS for photon energies up to about 2.5 eV among the materials investigated here. On the contrary, as both Cu and Ag have larger interband absorption edges (Cu: 2.2 eV, Ag: 4 eV) [1] due to d-bands, their JDOS curves do exist a cut-off photon energy (Cu: 2.23 eV, Ag: 3.52 eV). Though not shown here, gold has an interband absorption edge slightly higher than Cu [1] and exhibits a density of states distribution similar to that of Cu.

The advantage of using Al is also observed in the EDJDOS versus energy plots. While the JDOS determines the volume density of allowed transitions per unit energy for a given $h\nu$, it is how these allowed transitions distribute along the energy axis, which is described by the EDJDOS, that determines the number of photoexcited electrons (or holes) having sufficient energies to eventually overcome the Schottky barrier

***Corresponding author: Yin-Jung Chang,** Department of Optics and Photonics, National Central University, Taoyuan City, Taiwan, e-mail: yjchang@ncu.edu.tw
Ko-Han Shih, currently with CREOL, College of Optics and Photonics, University of Central Florida, Orlando, FL 32816, U.S.A.

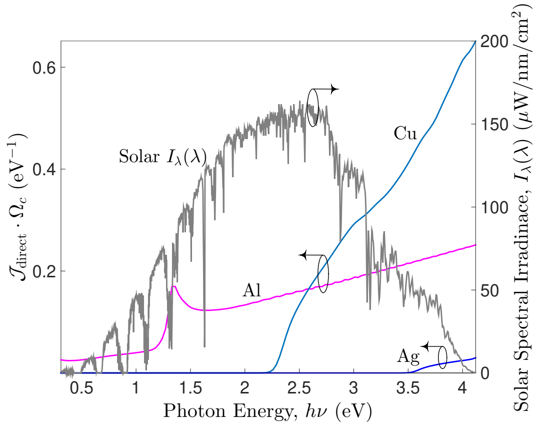

(a)

Fig. S1: Comparison of the joint density of states of Al, Cu, and Ag in one primitive cell (Ω_c: the volume of one primitive cell) as a function of photon energy $h\nu$ within the range of the solar irradiance spectrum [2].

upon emission. In the framework of direct transition, the EDJDOS $\mathcal{D}_{\text{direct}}(E_i, h\nu)$ is therefore written as [3]

$$\mathcal{D}_{\text{direct}}(E_i, h\nu) = \frac{1}{4\pi^3} \sum_{i,f} \int_{\text{FBZ}} f_F(E_i)[1 - f_F(E_f)]\delta\left(E_{f,\mathbf{k}} - E_{i,\mathbf{k}} - h\nu\right)\delta\left(E - E_{i,\mathbf{k}}\right) d^3\mathbf{k}, \tag{2}$$

where the second Dirac-delta function is introduced to select all transitions with an initial energy E_i. As observed in Fig. S2, since its band structure is free-electron-like, Al allows vertical transitions due to photons over a broad photon energy range. The peak EDJDOS is contributed mainly from the transitions near the W point where parallel bands exist.

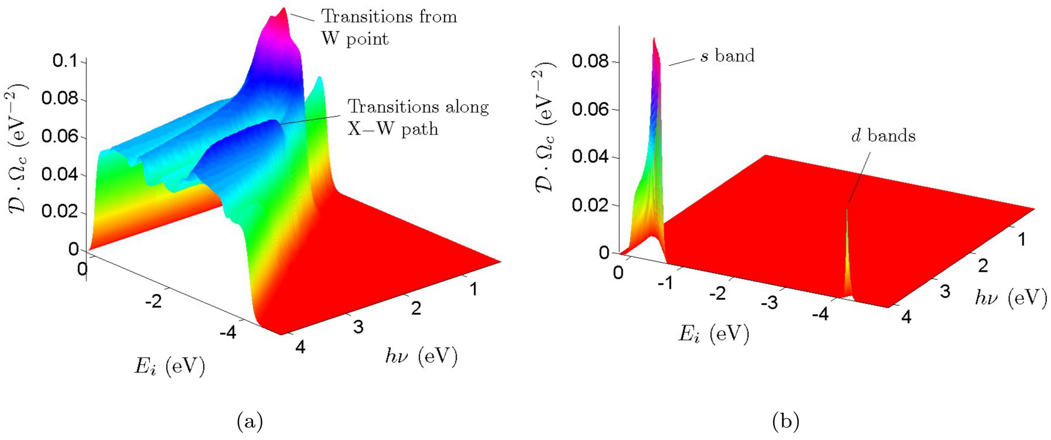

Fig. S2: Comparison of the energy distribution of joint density of states of Al and Ag in one primitive cell ($\mathcal{D} \cdot \Omega_c$) as a function of initial energy E_i and photon energy $h\nu$ within the range of the solar irradiance spectrum [2]. The temperature is at 300 K and the Fermi level is used as the reference energy at 0 eV.

On the contrary, for Ag, a photon energy threshold of about 3.52 eV is observed [Fig. S2(b)] below which the EDJDOS vanishes. The allowed vertical transitions occur mainly from the dispersive 5s band around the L point in the FBZ with $h\nu > 3.52$ eV, while the contributions from the 4d-bands become appreciably large only for $h\nu > 4$ eV, thus producing much less amount of hot electrons even in the visible

regime. As a result, Al may be more suitable for hot-electron-based photon energy conversion and we use Ag as the bottom layer material to minimize any reverse electron flow.

2 Connections between the incident polarization and field distributions underneath square prisms

To understand the connections between the incident polarization and the field distributions underneath square prisms (described in the main text), we take cases with incident polarization angles of 0°, 45°, and 90° as examples. In Fig. 5(a) of the main text, since the rotation angle of prism 3 is 0° and the incident wave is x-polarized (i.e. the polarization angle $\varphi = 0°$), GPP resonance underneath prism 3 produces a symmetric modal field concerning the x-axis (and the y-axis as well). Similarly, as the rotation angle of prism 1 is close to 45°, one diagonal line of symmetry is nearly parallel to the incident polarization, making the (nearly symmetric) field maxima appear in the vicinity of opposite vertices.

The same arguments apply to the field distributions across the mid-gap for $\varphi = 45°$ and $\varphi = 90°$ (i.e. y-polarized), as shown in Fig. S3. At $\varphi = 45°$ [Fig. S3(a)], since the incident polarization is parallel (nearly parallel) to prism 3's (prism 1's) one diagonal (non-diagonal) line of symmetry, strong field magnitudes are produced in the vicinity of (nearly parallel to) opposite vertices (sidewalls) of prism 3 (prism 1). For the y-polarized incidence [Fig. S3(b)], as φ is rotated by 90°, so are the field distributions underneath the prisms when compared to those at $\varphi = 0°$ [Fig. 5(a)].

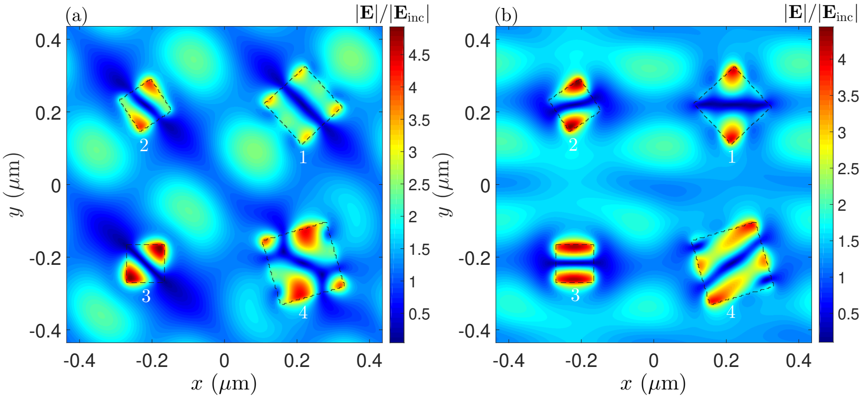

Fig. S3: Normalized electric field distribution (normalized to the incident $|\mathbf{E}_{\text{inc}}|$ field) across the mid-gap under normal incidence when the polarization angle (a) $\varphi = 45°$ and (b) $\varphi = 90°$ (i.e. y-polarized). The operating wavelength is $\lambda_0 = 627$ nm.

References

[1] M. Fox, *Optical Properties of Solids*, 1st ed., Oxford University Press, New York, 2007.

[2] Reference Air Mass 1.5 Spectra, ASTM G-173-03. [Online]. Available: https://www.nrel.gov/grid/solar-resource/spectra.html

[3] Y.-J. Chang and K.-H. Shih, "Solar energy conversion via internal photoemission in aluminum, copper, and silver: Band structure effects and theoretical efficiency estimates," *J. Appl. Phys.*, vol. 119, p. 183101, 2016.
